# Augmentation of cellular and humoral immune responses to HPV16 and HPV18 E6 and E7 antigens by VGX-3100

**DOI:** 10.1038/mto.2016.25

**Published:** 2016-11-30

**Authors:** Matthew P Morrow, Kimberly A Kraynyak, Albert J Sylvester, Xuefei Shen, Dinah Amante, Lindsay Sakata, Lamar Parker, Jian Yan, Jean Boyer, Christian Roh, Laurent Humeau, Amir S Khan, Kate Broderick, Kathleen Marcozzi-Pierce, Mary Giffear, Jessica Lee, Cornelia L Trimble, J Joseph Kim, Niranjan Y Sardesai, David B Weiner, Mark L Bagarazzi

**Affiliations:** 1 Inovio Pharmaceuticals, Plymouth Meeting, Pennsylvania, USA; 2 Unified Women’s Clinical Research, Winston-Salem, North Carolina, USA; 3 Johns Hopkins University School of Medicine, Baltimore, Maryland, USA; 4 The Wistar Institute, Philadelphia, Pennsylvania, USA

## Abstract

We have previously demonstrated the immunogenicity of VGX-3100, a multicomponent DNA immunotherapy for the treatment of Human Papillomavirus (HPV)16/18-positive CIN2/3 in a phase 1 clinical trial. Here, we report on the ability to boost immune responses with an additional dose of VGX-3100. Patients completing our initial phase 1 trial were offered enrollment into a follow on trial consisting of a single boost dose of VGX-3100. Data show both cellular and humoral immune responses could be augmented above pre-boost levels, including the induction of interferon (IFN)γ production, tumor necrosis factor (TNF)α production, CD8+ T cell activation and the synthesis of lytic proteins. Moreover, observation of antigen-specific regulation of immune-related gene transcripts suggests the induction of a proinflammatory response following the boost. Analysis of T cell receptor (TCR) sequencing suggests the localization of putative HPV-specific T cell clones to the cervical mucosa, which underscores the putative mechanism of action of lesion regression and HPV16/18 elimination noted in our double-blind placebo-controlled phase 2B trial. Taken together, these data indicate that VGX-3100 drives the induction of robust cellular and humoral immune responses that can be augmented by a fourth “booster” dose. These data could be important in the scope of increasing the clinical efficacy rate of VGX-3100.

## Introduction

The Human Papillomavirus (HPV) is well established as being an etiologic agent for cervical cancer, as well as being a major contributor linked to a diverse set of other cancers of mucosal tissue including vulvar, vaginal, penile, anal, oropharyngeal and in rare cases, respiratory.^1–8^ Although over 100 genotypes of HPV have been identified,^9^ types 16 and 18 are the most highly associated with the induction of cervical cancer^9,10^ as identification of one or both types is found in roughly 70% of cases.^10^ Additionally, HPV16 has been identified as the leading cause of HPV driven oropharyngeal squamous cell carcinoma with an estimated 90% prevalence.^11–13^ Treatments for HPV-associated precancerous and cancerous states vary based on disease and may fall anywhere from a “watch and wait” approach for the former to surgical intervention in addition to chemotherapy and radiation for the latter.^14–16^ There is currently no approved immunotherapeutic treatment for HPV-driven pathology.

In the cervix, the intraepithelial lesion that precedes virtually all squamous cervical cancers, cervical intraepithelial neoplasia 2/3 (CIN2/3), is treated with surgical excision.^14,16^ However, surgical resection does not lead to complete elimination of the virus in cervical tissues in all cases, resulting in persistent HPV infection of the type initially responsible for the disease state.^17,18^ As persistent HPV infection is a significant risk for disease recurrence, HPV persistence after resection is also a significant risk factor for disease recurrence.^15,17,19^ Thus, viral clearance represents an important and desirable component of effective treatment of advanced dysplasia that surgical intervention may not be best suited for. As immune-mediated control and elimination of chronic viral infections are associated with CD8+ T cell responses,^20–23^ an immunotherapy able to generate HPV-specific T cells that show long-term persistence and can migrate to cervical tissues and mediate clearance of virus would be a useful tool for prevention of disease recurrence in patients with persistent infection. Moreover, the ability to benefit from additional treatments of such an immunotherapy would prove useful in this endeavor.

We have previously reported data from a phase 1 study of tolerability, safety, and immunogenicity of an HPV16/18 candidate DNA vaccine, VGX-3100, delivered by intramuscular (IM) injection followed by *in vivo* electroporation (EP), in women who had undergone an excisional procedure for intraepithelial HPV disease.^24^ We have also reported the results from our phase 2 double-blind randomized placebo-controlled efficacy study of VGX-3100 in which we noted statistically significant rates of regression of HPV16/18-positive CIN2/3 and clearance of HPV16/18, which were statistically associated with an immunological response.^25^ Here, we describe a follow-on Phase I trial in which thirteen of the eighteen subjects enrolled in the original Phase I study were administered a single boost of 6 mg VGX-3100 followed by EP in order to study the ability of previously generated HPV16- and HPV18-specific immune responses to be further boosted. Immune responses measured after the boost revealed an augmented humoral response, an increase in cytokine expression from both the CD4+ and CD8+ T cell compartments and an increase in the expression of lytic proteins within HPV-specific CD8+ T cells. These responses were similar in magnitude and quality to those observed in our phase II double-blind placebo-controlled efficacy study of VGX-3100 (ref. 25). Additionally, in the current study, we determined the diversity of TCRs in T cells isolated from subject-matched peripheral blood and cervical tissue samples. Clonally expanded TCRs that appeared after vaccination, and were also present in the cervix suggest putative HPV-specific TCRs. These data suggest that VGX-3100 elicits humoral responses and cellular responses with a CTL phenotype, and that both have the ability to be boosted by additional administrations. Taken together with efficacy data generated from our phase 2 study, the data presented here suggest that VGX-3100 generates immune responses which may be critical in preventing recurrence of disease as well as the opportunity for additional dosing to further augment responses suggesting it is an attractive alternative to surgical intervention for advanced cervical dysplasia.

## Results

### Study subjects

Screening evaluations were performed within 30 days prior to study enrollment on Day 0. If the screening assessments were performed within 7 days of dosing on Day 0, a single assessment could count as both the screening and Day 0 evaluations, however, pregnancy testing had to be repeated if screening and Day 0 assessments were performed greater than 48 hours apart.

The Month 9 visit of the HPV-001 study could count as the screening visit for the HPV-002 study, provided that all of the necessary evaluations for both visits were performed. A total of 18 subjects were identified for screening from the HPV-001 study, of which 13 were eligible for HPV-002 and 13 provided written informed consent, and were enrolled to receive 6 mg of VGX-3100 followed by EP, regardless of the dose that they received in the HPV-001 study. Three subjects were not eligible due to pregnancy, one was not given the option to participate in HPV-002 due to protocol noncompliance, and one subject declined due to shoulder pain. All 13 subjects received 6 mg of VGX-3100, and all except one completed the follow-up visit (Month 6). The mean age of the study population was 30.5 years (range: 20–42 years).

### Safety and tolerability of VGX-3100 followed by electroporation

#### Injection site reactions.

Injection site reactions were evaluated within 30 minutes after EP and on Day 7 postdose. Overall, injection of 6.0 mg of VGX-3100 followed by EP was well-tolerated. No severe or potentially life-threatening reactions were noted during the course of the study. Local postadministration injection site reactions were evaluated for severity by “Grade” on Day 0 at 15 minutes post-EP and on Day 7 postdose. “Grade” was defined as Grade 1 = “mild”, Grade 2 = “moderate”, Grade 3 = “severe,” and Grade 4 = “potentially life threatening.” One or more of the local postadministration injection site reactions (pain, tenderness, redness, and/or swelling) were experienced by each of the 13 subjects. No grade 3 or 4 adverse events (AEs) or serious adverse events (SAEs) were observed during the study (Supplementary Table S1).

#### Treatment emergent adverse events.

No deaths or serious adverse events were reported and no adverse events led to withdrawal from the study. There were also no Grade 3 or Grade 4 adverse events reported. Overall, eight subjects (62%) reported any treatment emergent AEs with the highest severity as “moderate” for one subject, and “mild” for five subjects. All were determined to be not related or not likely related to VGX-3100.

### Boosting with VGX-3100 augments HPV-specific humoral immune responses and production of IFNγ generated by VGX-3100 priming

**Figure 1:**
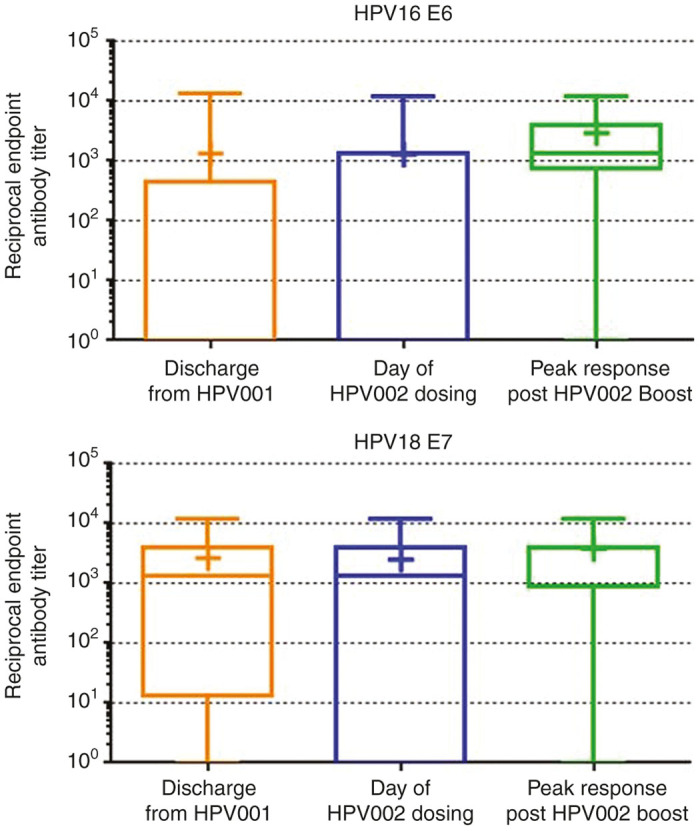
Boosting with VGX-3100 augments humoral immune responses. Box and whisker plots show endpoint ELISA titers against HPV16 E6 (top panel) and HPV18 E7 (lower panel) after discharge from HPV001, on the day of entry into HPV002 and following the boost of VGX-3100 administered in HPV002. Bars indicate minimum and maximum values observed, lines within the box indicate median response, plus signs indicate mean response. HPV, Human Papillomavirus.

In our previously reported phase 1 dose-ranging trial (HPV-001) consisting of a three-dose regimen of 0.6 to 6 mg VGX-3100, we identified robust *de novo* cellular immune responses generated after immunization that persisted out to 6 months following the third dose.^24^ Upon establishing this, we began analysis of the impact of a single 6.0 mg boost with VGX-3100 had on that established HPV-specific immune response. To that end, we performed ELISA and IFNγ ELISpot in order to assess the humoral and cellular compartments, respectively. Observation of humoral responses following the boost revealed that the median endpoint titer against HPV16 E6 and HPV18 E7 proteins at entry of HPV002 (0 and 1:1,350, respectively) were boosted by the administration of one dose of VGX-3100 to a median titer of 1:1,350 and 1:4,050, respectively (Figure 1). Responses against the other two antigens did not benefit from the boost (Supplementary Figure S1). In total, 10 of the 13 subjects (76.9%) showed an increase in endpoint titer against HPV16 E6 and 7 of the 13 (53.9%) showed an increase in endpoint titer against HPV18 E7 when compared to the titer at enrollment in HPV-002. The remaining subjects showed a sustained titer between timepoints with no decrease in titer. These data further highlight the persistence of response generated by VGX-3100 and suggest the boost had a positive impact on subject antibody titers (Figure 1).

**Figure 2:**
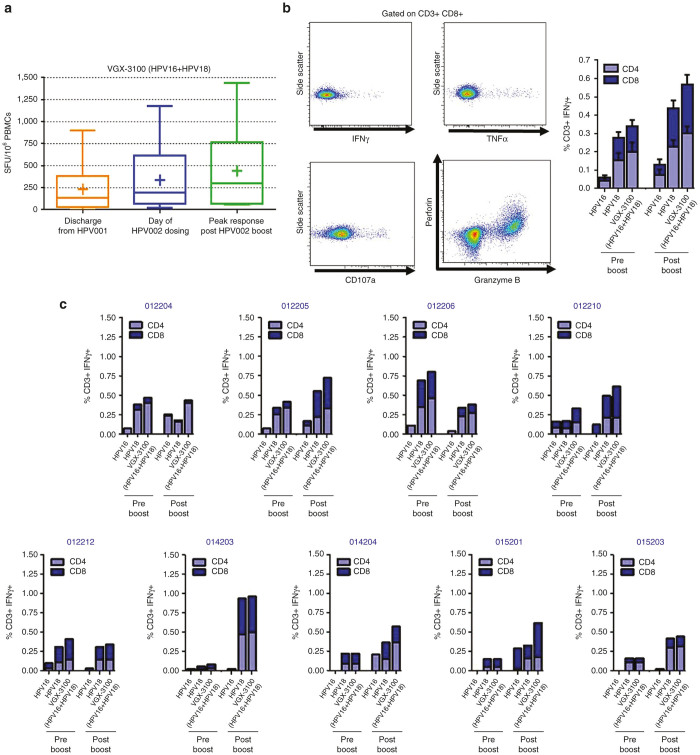
Boosting with VGX-3100 augments interferon-γ production. (**a**) Box and whisker plots show Interferon-γ ELISpot responses against VGX-3100 antigens after discharge from HPV001, on the day of entry into HPV002 and following the boost of VGX-3100 administered in HPV002. Bars indicate minimum and maximum values observed, lines within the box indicate median response, plus signs indicate mean response. (**b**) Representative staining of Interferon-γ, tumor necrosis factor-α, CD107a, Granzyme B and Perforin expression (left panels) as well as cumulative Interferon-γ production from the CD4+ and CD8+ T cell compartments across the study pre- and postboost (right panel). (**c**) Individual patient data for contribution of Interferon-γ production from CD4+ and CD8+ T cells. HPV, Human Papillomavirus.

Analysis of IFNγ ELISpot responses show that the median response at entry of HPV002 (195 SFU/10^6^ Peripheral Blood Mononuclear Cells (PBMCs)) was boosted by 1.5-fold after the administration of one dose of VGX-3100 to a median magnitude of 297 SFU/10^6^ PBMCs (Figure 2a). In total, 8 of the 12 subjects (66.7%) with evaluable sample showed an increase in SFU when compared to their magnitude at enrollment in HPV002, suggesting that the single boost with VGX-3100 imparted a beneficial influence on the HPV-specific cellular immune response for the majority of the subjects in the study.

In order to determine the relative contributions of CD4+ T cells and CD8+ T cells to the production of IFNγ, we employed flow cytometric analysis (Figure 2b). Examination of HPV-specific responses across the study population revealed that HPV16 and HPV18-specific IFNγ production was increased after administration of the boost in both the CD8+ and CD4+ T cell compartments, with the former increasing from 0.20 to 0.30% in total magnitude and the latter increasing nearly twofold from 0.14 versus 0.27%, amounting to a cumulative increase of over 1.5-fold in the T cell compartment as a whole (0.34 versus 0.57%, respectively, Figure 2b). Analysis on a subject-by-subject basis for whom sufficient sample was present revealed six of the nine subjects (66.7%) had an increase in HPV-specific IFNγ production from one or both T cell compartments, with two subjects showing increased production in the CD8+ compartment only and the remaining four subjects showing increased IFNγ production from both T cell compartments (Figure 2c). Magnitudes of IFNγ production in these six subjects ranged from 0.18 to 0.50% in the CD4+ compartment and 0.13 to 0.47% in the CD8+ T cell compartment (Figure 2c). Observation of the relative contributions of response broken out by T cell maturation status reveals that putative Effector Memory cells (CD45RO+/CCR7-/CD27-) were the primary producers of HPV16- and HPV18-specific IFNγ in the CD4+ T cell compartment with Effectors (CD45RO-/CCR7-CD/27-) contributing slightly less and Transitional (CD45RO+/CCR7-/CD27+) and Central Memory (CD45RO+/CCR7+/CD27+) populations contributing the least (Supplementary Figure S2). The CD8+ T cell compartment exhibited a different pattern in which Effectors (CD45RO-/CCR7-/CD27-) were the main contributors, followed by Effector Memory (CD45RO+/CCR7-/CD27-), Transitional Memory (CD45RO+/CCR7-/CD27+), and Central Memory (CD45RO+/CCR7+/CD27+), respectively (Supplementary Figure S2).

### Boosting with VGX-3100 expands proinflammatory cytokine production from T cells and expands polyfunctional CTL phenotypes

**Figure 3:**
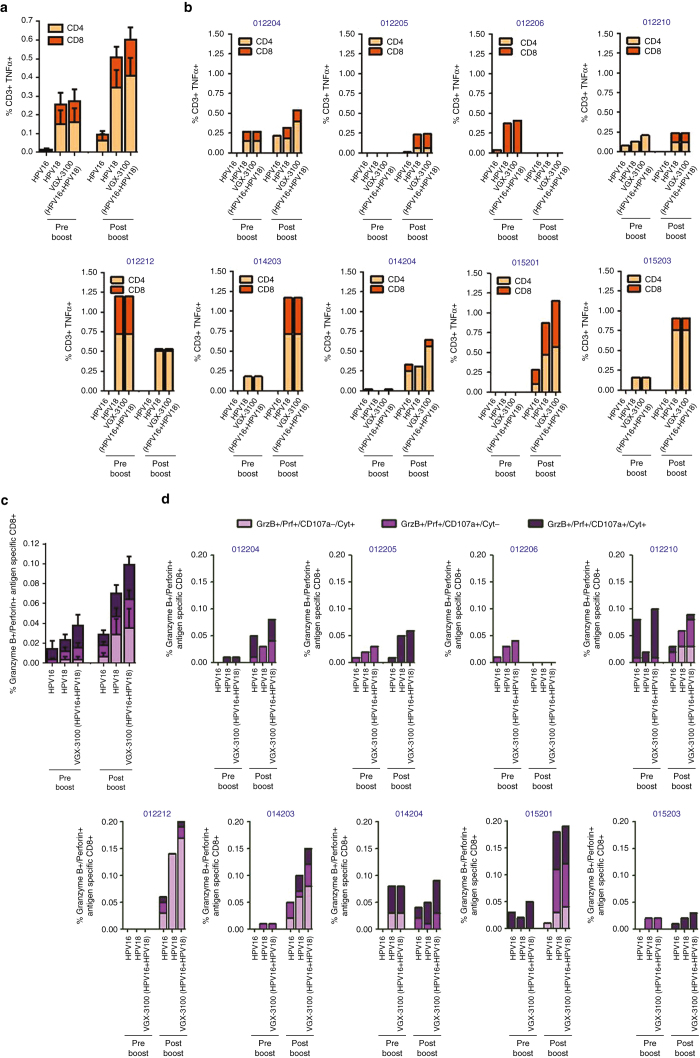
Boosting with VGX-3100 augments production of cytokines and lytic proteins associated with cytotoxicity. (**a**) Cumulative TNFα production from the CD4+ and CD8+ T cell compartments across the study population pre- and postboost. (**b**) Individual patient data for contribution of TNFα production from CD4+ and CD8+ T cells. (**c**) Cumulative cytokine and lytic protein production from the CD8+ T cell compartment across the study population pre- and post-boost, where “Cyt” stands for “Cytokine” being either IFNγ or TNFα. (**d**) Individual patient data for cytokine and lytic protein production from the CD8+ T cell compartment.

As the production of IFNγ is a strong measure of a Th1-associated immune response but is not always directly associated with lytic activity,^24,26,27^ we further analyzed T cell responses driven by administration of a boosting dose of VGX-3100 in regards to the production of TNFα—a cytokine which exhibits both a proinflammatory effect as well as the ability to drive cell death in tumor tissues.^28,29^ Examination of HPV-specific responses across the study population revealed that HPV16- and HPV18-specific TNFα production was markedly increased after administration of the boost in both the CD8+ and CD4+ T cell compartments, with the former increasing nearly twofold (0.11 versus 0.19%) and the latter increasing more than 2.5-fold from pre- to postboost (0.16 versus 0.41%, Figure 3a) amounting to a cumulative increase of over twofold in the T cell compartment as a whole (0.27 versus 0.60%, respectively). Analysis on a subject-by-subject basis revealed seven of the nine evaluable subjects (77.8%) had an increase in HPV-specific TNFα production from one or both T cell compartments, with one subject showing increased activity in the CD4+ compartment only, one subject showing increased production in the CD8+ compartment only and the remaining five subjects showing increased TNFα production from both T cell compartments (Figure 3b). Magnitudes of TNFα production in these subjects ranged from 0.06 to 0.76% in the CD4+ compartment and 0.08 to 0.58% in the CD8+ T cell compartment (Figure 3b). Observation of the relative contributions of response broken out by maturation status reveals a slightly different pattern as compared with IFNγ production. Both Effector (RO-/R7-/27-) and Effector Memory (RO+/R7-/27-) were the primary producers of HPV16 and HPV18-specific TNFα in both the CD4+ and C8+ T cell compartments, with minor production stemming from the Transitional (RO+/R7-/27+) and Central Memory (RO+/R7+/27+) populations (Supplementary Figure S3).

Apoptotic death of tumor cells can be mediated through the immune system via mechanisms other than TNFα, and in particular may stem in large part from the synthesis and release of lytic proteins such as granzyme B and perforin.^30–32^ Therefore, we also assessed the CD8+ T cell compartment for HPV-specific concurrent expression of these lytic proteins with varying combinations of IFNγ, TNFα and CD107a—the latter being an established marker of cytolytic degranulation.^33^ Using these markers, we focused on three distinct CD8+ T cell profiles: (i) those that co-expressed granzyme B and perforin concurrent with a cytokine (IFNγ, TNFα, or both) in the absence of CD107a (termed GrzB+Prf+/CD107a-/Cytokine+), (ii) those that coexpressed granzyme B and perforin concurrent with CD107a but in the absence of a cytokine (termed GrzB+Prf+/CD107a+/Cytokine-), and (iii) those that were positive for granzyme B, perforin, CD107a and at least one cytokine (termed GrzB+Prf+/CD107a+/Cytokine+). The first described population would constitute CD8+ T cells that were HPV specific and had lytic potential but did not degranulate within the 6-hour window of the assay (as evidenced by being CD107a-). Such cells would likely not be capable of direct lysis within that narrow timeframe, but would harbor the correct lytic proteins to mediate this type of cell death if prolonged stimulation allowed for them to degranulate. Moreover, expression of TNFα from these cells could mediate receptor/ligand-mediated lysis and the production of IFNγ could aid in sensitizing tumor cells to receptor-mediated lysis.^34–36^ The second population would constitute those CD8+ T cells that were HPV specific and harbored lytic potential as well as the ability to degranulate as evidenced by being positive for CD107a. The final population expresses all markers consistent with a high-functioning population of CTLs, which have the capability of killing tumor cells by multiple mechanisms including direct lysis via lytic proteins, receptor/ligand-mediated lysis and sensitizing tumors to lysis by lytic granules. All three populations require concomitant expression of at least three antigen-specific regulated markers, thereby enforcing a requirement for polyfunctionality in order to be considered for analysis. Examination of these phenotype’s across the study population revealed that total HPV16 and HPV18-specific activity was augmented by administration of the boost over two-fold as compared with preboost activity (0.04 versus 0.10%, respectively, Figure 3c). Analysis on a subject-by-subject basis revealed seven of the nine evaluable subjects (77.78%) had an increase in HPV-specific polyfunctional CTL phenotypes, with four of the seven showing changes restricted to only one of the above described phenotypes and the remaining three showing activity in all three phenotypes (Figure 3d). Total magnitude of response taking all three phenotypes into account in subjects with detectable changes after the boost showed a range of 0.03 to 0.20% (Figure 3d). These data together suggest that boosting subjects with VGX-3100 can augment HPV-specific T cell profiles associated with enhanced antigen-specific killing activity through multiple mechanisms including receptor/ligand and lytic degranulation.

### Genes associated with inflammatory, cytotoxic, and effector responses are regulated in an antigen-specific manner in PBMCs after a booster dose of VGX-3100

**Figure 4:**
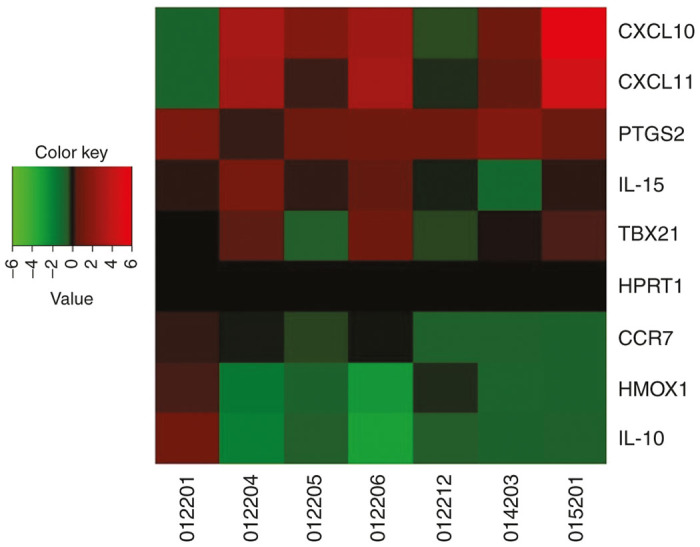
Genes associated with inflammatory, cytotoxic and effector responses are regulated in an antigen-specific manner in PBMCs after boosting with VGX-3100. A heatmap showing individual patient responses for eight gene transcripts differentially regulated upon antigenic stimulation. A color key for upregulation or downregulation of a given transcript is included where green indicates downregulation of a transcript and red indicates upregulation. Black coloring indicates no change based on antigen stimulation. HPRT1 is has been included as a control housekeeping gene.

Genetic analysis of PBMCs using Reverse Transcriptase quantitative polymerase chain reaction (RTqPCR) affords the opportunity to examine various parameters of an inflammatory response in an exploratory manner, broadening the possibility of identifying immune signatures from cells that had either been unstimulated or stimulated with HPV antigens encoded by VGX-3100. Comparison of these samples allowed us to identify a set of genes that were differentially regulated based on HPV16/18-specific activation. Results of this analysis are presented in a heatmap in Figure 4 for the seven patients from whom we had evaluable sample. Five gene transcripts associated with a proinflammatory response were found to be upregulated in the majority of patients tested after antigenic stimulation: CXCL10, CXCL11, PTGS2, IL-15, and TBX21. CXCL10 and -11 are also known as Interferon Gamma Inducible Protein 10 and 9, respectively (IP10 and IP9) and are activated downstream of Interferon Gamma production, a hallmark of an inflammatory Th1 response.^37^ PTGS2 is also known as cyclooxygenase-2 (COX-2) and is a well established proinflammatory molecule.^38^ IL-15 (interleukin 15) drives the expansion and activation of CD8+ T cells, including the induction of the synthesis of lytic proteins necessary for an effective cytotoxic response.^39^ Additionally, TBX21 (also known as Tbet) is a transcription factor known to directly activate transcription of Interferon-γ and is associated with increased antigen-specific CD8+ T cell cytotoxicity.^40^ Moreover, three transcripts were found to be downregulated in patient PBMCs in an antigen-specific manner: CCR7, IL-10, and HMOX1. CCR7 is a chemokine receptor that is well established as identifying cells that will home to and remain in the lymph nodes in a Central Memory or Naive phenotype. Thus its downregulation is suggestive of an antigen-specific shift towards an effector phenotype. IL-10 (interleukin 10) is a known suppressive factor for the induction of an inflammatory and cytotoxic response, thus its downregulation after antigenic stimulation aids in ensuring these responses are not turned off. HMOX1 encodes for heme oxygenase 1, which has been shown to mediate the anti-inflammatory effect of IL-10 (ref. 41), thus it’s downregulation in concert with IL-10 supports the notion of a lack of activation in this suppressive pathway. Taken together, the modulation of these genes in an antigen-specific manner suggests that PBMCs exhibit a variety of proinflammatory and cytotoxic hallmarks after receiving a boost with VGX-3100.

### Boosting with VGX-3100 supplements cosynthesis of granzyme B and perforin in activated HPV-specific CD8+ T cells generated by VGX-3100 priming

**Figure 5:**
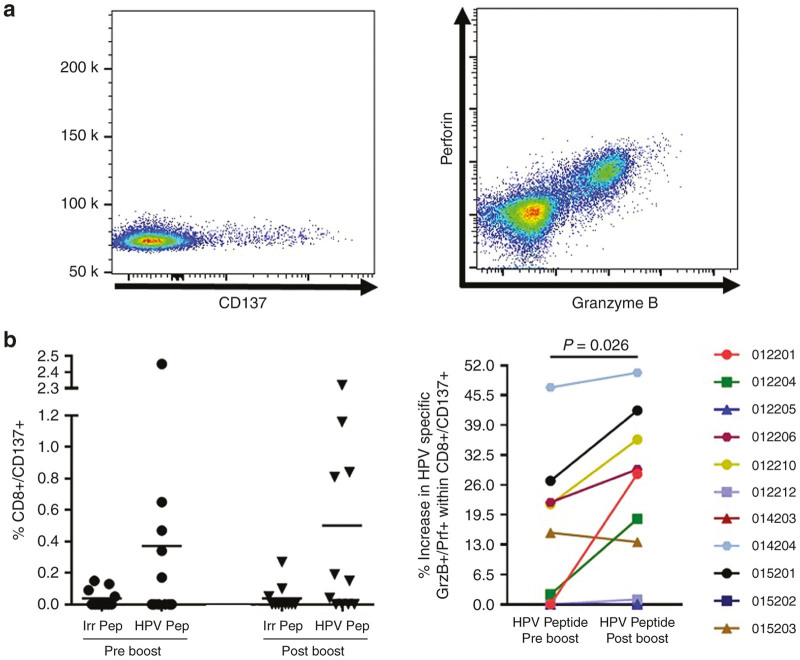
Additional dosing of VGX-3100 increases lytic protein content of activated (CD137+) CD8+ T cells. (**a**) Representative staining of the activation marker CD137 (also known as 4-1BB) as well as Granzyme B and Perforin in CD8+ T cells. (**b**) Antigen-specific expression of CD137 on patient CD8+ T cells in response to extended stimulation with VGX-3100 antigens prior to and following the boost (left panel). The right panel displays data regarding cosynthesis and expression of Granzyme B and Perforin within the activated (CD137+) CD8+ T cell subset in response to VGX-3100 antigens (Human Papillomavirus (HPV) peptide stimulation). Irr Pep = Irrelevant Peptide. HPV Pep = peptides spanning HPV16 and HPV18 E6 and E7

Chronic viral infections are frequently associated with immune exhaustion or senescence in the context of immune reactivity to viral antigens.^42–44^ Although mechanisms behind these phenomena vary, a heavy and prolonged antigenic burden on the cellular arm of the immune system is thought to play a role in this immune incompetence. Study of cellular immune responses from our previous phase 1 dose-ranging trial demonstrated that HPV-specific CD8+ T cells generated by VGX-3100 were able to activate, remain active, and synthesize lytic proteins during a prolonged antigenic stimulation (120 hours).^24^ We also noted this ability in our phase 2 study and established a statistical association between this phenomenon and clinical efficacy.^25^ Thus, this type of profile is believed to be key for an effective CD8+ T cell response capable of eliminating a chronic viral infection and is an important hallmark of an appropriate immune response generated by VGX-3100. In the current study, we sought to determine whether a single booster dose of VGX-3100 augmented the frequency of HPV-specific activated CD8+ T cells as well as whether or not any notable increase in cosynthesis of granzyme B and perforin occurred. To that end, we performed a prolonged antigenic stimulation (120 hours) using HPV peptide and irrelevant control peptide (Ovalbumin) on subject PBMCs that were isolated prior to and after the boost and assessed activation status and lytic protein synthesis by flow cytometry (Figure 5a). CD137 (also known as 41BB^45,46^) was used as a marker of CD8+ T cell activation. CD137 has been established by our group and others as an accurate marker of antigen-specific activation^45,46^ and its expression is linked with a functional tumor-infiltrating lymphocyte profile,^47^ making it particularly relevant in the context of advanced dyplasia and cancer driven by HPV infection. Analysis of the data generated by this assay reveals that the boost did not appreciably increase the frequency of total HPV-specific activated CD8+ T cells as determined by CD137 expression (Figure 5b, left panel) which was in part due to pre-existing responses noted prior to the boost. As nonspecific CD8+ T activation by irrelevant (control) peptide was low or nonexistent (Figure 5b, left panel) the presence of pre-existing HPV-specific responses within the assay are likely tied to initial dosing with VGX-3100 in the previous trial. Further examination of HPV-specific CD8+ T cells showed that while no major change in frequency was noted, a statistically significant shift in HPV-specific coexpression of granzyme B and perforin was present (*P* = 0.026, Figure 5b, right panel), suggesting that the boost augmented the production of lytic effector molecules within these activated HPV-specific CD8+ T cells. Taken together, these data suggest that initial dosing with VGX-3100 drives persistent CD8+ T cell responses and that a boost is able to impart a statistically significant increase in the ability to cosynthesize lytic proteins associated with antigen-specific lysis of virally infected and tumor cells.

### Boosting with VGX-3100 expands putative HPV-specific T cells localizing to cervical tissues

Following our observation of increased HPV-specific T cell responses in the peripheral blood after a boost with VGX-3100, we were next interested in determining if HPV-specific T cells were able to be detected in the cervical mucosa and if the frequencies of these sequences increased after the boost. Previous studies in humans have shown the ability of intramuscular immunization to elicit cellular responses at cervical sites during HPV infection^48^ as well as elicit both cellular and humoral immune responses at mucosal sties in the absence of viral infection.^49^ As cervical biopsies were not performed during the trial, we did not have any tissue available for immunohistochemistry for direct visualization of infiltrating lymphocytes. However, high-throughput TCR sequencing of the hypervariable complementarity-determining region 3 (CDR3) of the TCR β chain^50^ has previously been utilized to identify HPV-specific clonal expansions in the cervical tissue.^48^ In the current study, samples from the cervix were collected in the form of Digene swabs both before and after the boost was administered. We thus performed TCR sequencing from digene samples in order to determine if a single boost of VGX-3100 influenced putative TCR signatures in cervical tissue. To that end, paired cervical specimens and PBMC samples from a subset of immunized patients (*n* = 6) both pre- and postboost were obtained and allocated for TCR sequencing.

As cervical samples were not collected in a manner allowing for restimulation, these swabs were processed without any *ex vivo* expansion. Initial analysis revealed that the frequency of numerous TCR sequences was increased in the postboost cervical swab when compared to the same patient’s preboost sample (Supplementary Figure S4). To determine whether these expanded TCR clones were putative HPV-specific sequences, we used patient matched PBMC samples as a reference point, reasoning that we could identify HPV-specific TCR signatures on a per-patient basis by performing antigen-specific PBMC expansion followed by sequencing. To that end, we expanded peripheral HPV-specific T cells from the pre- and postboost timepoints by stimulating PBMCs for 5 days with HPV peptides followed by TCR sequencing. TCR clones that were found in greater frequency in the postboost expanded PBMCs as compared to the preboost were designated as putatively HPV specific based on their antigen-specific expansion. To help confirm that the TCR sequences identified in this way truly were HPV specific, we utilized PBMCs from three different cord blood samples as a negative control, reasoning that clonal expansion seen in these samples after HPV peptide stimulation would constitute nonspecific amplification. One of three cord blood samples contained four sequences that were elevated after HPV peptide stimulation (Supplementary Table S2) and these sequences were subsequently excluded from further analyses.

**Figure 6:**
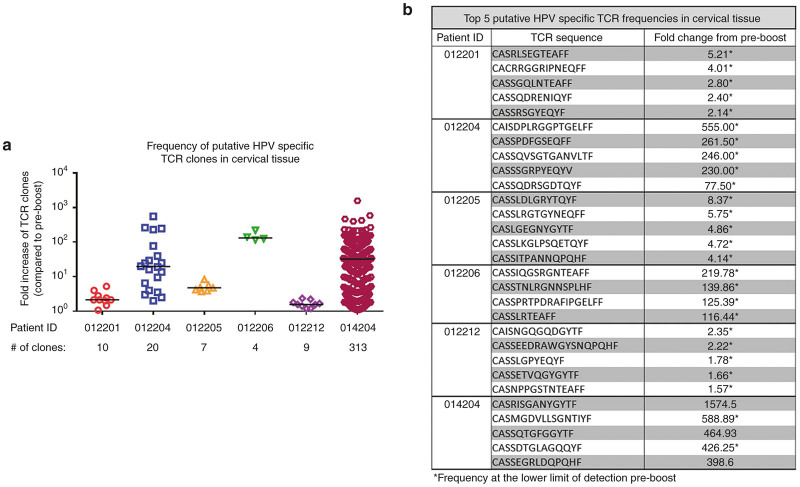
Putative Human Papillomavirus (HPV)-specific T cell receptor clones localize to cervical tissue following a boost with VGX-3100. (**a**) Fold changes in the frequency of putatively HPV-specific TCR clones in cervical tissue are graphed comparing pre- and postboost frequencies on a per-patient basis. T cell receptor sequences were identified as putatively HPV-specific using control samples isolated from peripheral blood as well as cord blood samples as a negative control. (**b**) The top five putative HPV-specific TCR sequences in cervical tissue are listed on a per patient basis.

We next analyzed the sequencing runs from cervical samples for the presence of HPV-specific TCR signatures as we had defined above. Results of the analysis reveal that all patients assayed showed an expansion of HPV-specific TCR clones in the cervical tissue following a boost with VGX-3100 (Figure 6a). The degree of expansion of these clones in the cervical tissue varied from patient to patient, with some expansions reaching nearly 600-fold when comparing preboost and postboost timepoints. The five sequences with the greatest fold-increase postboost for each patient are listed in Figure 6b and additional sequences are listed in Supplementary Table S4. Interestingly, we found that the majority of TCR sequences that increased postboost did so at a much greater frequency in the cervix as compared to the periphery (Supplementary Tables S3 and S4), suggesting the ability of these T cells to move out of circulation into cervical tissue. Taken together, these data indicate that boosting with VGX-3100 is able to expand HPV-specific T cells in the periphery and that these cells have the ability to expand and home to the cervical mucosa, a necessary component of a T cell response whose aim is to eliminate viral infection in these tissues.

## Discussion

Persistent infection with a high risk HPV types such as HPV16 or HPV18 is strongly correlated with an increased risk of adenocarcinoma of the cervix, making elimination of infection of paramount importance in the prevention of this cancer.^51–53^ High-grade cervical dysplasia is treated by surgical excision in the form of conization or loop electrosurgical excision procedure (LEEP^14–16,53^). While these procedures are often effective, they are not a targeted approach to elimination of HPV infection. Indeed, analysis of patients at varying intervals after LEEP procedures reveal that many remain HPV positive and the mean recurrence rate of CIN2/3 after LEEP is roughly 10%.^17–19^ The development of a method of treatment of high-grade dysplasia that leads to the complete resolution of infection would not only treat and regress dysplastic lesions caused by HPV infection, but would also prevent recurrence of dysplasia. The patient’s own immune system presents itself as the natural choice for the development of such a treatment, as it has been previously established that the induction of an HPV-specific immune response, particularly a cellular response, is often identified in the context of resolution of HPV-driven pathology and elimination of viral infection.^22,23^ Moreover, the establishment of immunological memory presents itself as an attractive concept for this purpose as a durable cellular immune response that was capable of mediating regression of an initial infection should be able to control and eliminate future HPV infections of the same type. The ability to boost this response would aid in further ensuring that immune system was capable of controlling HPV-driven pathology and eliminating infection. To this end, we have designed an immunotherapy based on the induction of HPV-specific T cell responses generated via the use of plasmid DNA delivered by *in vivo* electroporation.

We report here data generated from a follow-on phase 1 trial in13 women who had previously received VGX-3100, an HPV16/18 candidate DNA immunotherapy for treatment of advanced cervical intraepithelial neoplasia. Review of safety data showed no SAEs or grade 3 or 4 AEs, consistent with our findings from the phase 1 dose ranging study.^24^ The immunological data reported here are the first data generated in regards to the ability to boost responses driven by VGX-3100 with subsequent dosing. Analyses conducted after administration of a single 6 mg boost of VGX-3100 revealed a therapy-driven increase in the production of HPV-specific antibodies as well as markedly increased cytokine production from both the CD4+ and CD8+ T cell compartments in the form of IFNγ and TNFα (Figures 2 and 3). Investigation of CTL phenotypes such as expression of lytic proteins (granzyme B, perforin), degranulation markers (CD107a), and activation markers on CD8+ T cells (CD137) additionally revealed an increase in the frequency of HPV-specific putative CTLs as well as in the ability of activated HPV-specific CD8+ T cells to cosynthesize granzyme B and perforin (Figures 3 and 5). Moreover, genetic hallmarks of a proinflammatory response were noted including the regulation of gene transcripts that are influenced by the production of IFNγ as well as a downregulation of gene transcripts associated with the repression of an inflammatory response (Figure 4). Of great interest was the finding that boosting with VGX-3100 not only influenced peripheral immune responses, but also the frequency of putative HPV-specific TCR signatures in cervical tissues (Figure 6). This finding falls in line with the immune signatures noted in our phase 2b efficacy study in which we noted a statistically significant influx of CD8+ T cells into the cervical mucosa in patients who exhibited regression of CIN2/3 lesions.^25^ Taken together, these data suggest that VGX-3100 induces durable immune responses and that boosting patients previously dosed with VGX-3100 with a single 6.0 mg injection is safe, well tolerated, and capable of further expanding these responses.

The data presented here are encouraging in regards to treatment of advanced cervical dysplasia on multiple fronts: the ability to boost the magnitude of response by administering an additional dose of VGX-3100 opens an avenue for the continued use of this immunotherapy in the treatment of patients with high-grade dysplasia, allowing for the opportunity of immune mediated lesion regression and viral elimination in a higher frequency of patients. Moreover, when taken in the context of our phase 2b results in which greater than 80% of patients treated with VGX-3100 whose CIN2/3 regressed did so in the form of regression to normal tissue, a pattern is established in which robust HPV-specific cellular immune responses are durable, can be boosted and are ultimately statistically associated with histological regression. These data and attributes of VGX-3100 make it a very attractive alternative to surgical intervention for the treatment of advanced cervical dysplasia driven by HPV16 or HPV18 infection.

## Materials and Methods

### Study participants

HPV-002 was a phase 1, open-label study to evaluate the safety, tolerability, and immunogenicity of a fourth, 6 mg dose of VGX-3100 (DNA plasmid encoding E6 and E7 proteins of HPV types 16 and 18) followed by *in vivo* electroporation (EP) in adult women who have been previously treated with three doses of VGX-3100 containing 0.6, 2, or 6 mg of DNA per dose. The study was conducted at three centers in the United States (clinicaltrials.gov registration NCT01188850). The study protocol was approved by a central Institutional Review Board (Integreview) and the protocol was adhered to by following the guidelines of Good Clinical Practices and the Declaration of Helsinki. Written informed consent was obtained prior to participation in the HPV-001 study and again, prior to participation in the HPV-002 study. The subjects had to have successfully enrolled and completed all study procedures and follow-up in the HPV-001 study. Adult women deemed eligible for participation were between 18–46 years of age, inclusive, had a diagnosis of CIN 2 or 3 resulting in a postsurgical (including LEEP and conization) or ablative treatment while under a physician’s care (according to American Society for Colposcopy and Cervical Pathology guidelines). Women of child bearing potential could not be pregnant or nursing and agreed to remain sexually abstinent, use medically effective contraception, or have a partner who is sterile from time of enrollment to study discharge. Each subject was required to have a serum pregnancy test during screening with a negative result prior to enrollment. A negative spot urine pregnancy test was required prior to each dose.

Major exclusionary criteria included the subject not being able to participate in a study with an investigational compound or device (other than VGX-3100) within 30 days of signing informed consent, and would be excluded if she had an active infection with herpes simplex virus.

### Study design

Subjects that were previously enrolled in the HPV-001 study and received three doses (containing 0.6, 2, or 6 mg of DNA/dose) of VGX-3100 (at Day 0, Month 1, and Month 3), and who consented to participate in the HPV-002 study were given a 1.0 ml intramuscular injection containing 6 mg of VGX-3100 in the deltoid followed immediately by IM EP with the CELLECTRA-5P device. The boost was administered no earlier than 6 months following the third dose (Month 3 of the first trial) in the HPV-001 study, and subjects were followed for 6 months after their fourth dose.

### Safety assessment

Subjects were monitored for fever, local (injection site reactions) and clinical AEs, and SAEs. The injection site was examined prior to and within 30 minutes after EP on the day of treatment and again at approximately 7 days later. PAP smears were also performed at Day 0 (prior to dosing) and at the 6-month follow-up visit. Additional safety assessments included physical examination at screening and discharge, vital signs, pre- and postdose ECG, and evaluation of clinical laboratory tests (data not shown). Samples for virologic assessments will were obtained using ThinPrep test kits. The ThinPrep sample was sent to a central laboratory to be analyzed for the presence of HPV high-risk genotypes and if positive, were reflexed to HPV genotyping via polymerase chain reaction (PCR) assay. Cervical cytology samples were collected to test for the presence of HPV and genotyping in subjects before and after administration of VGX-3100. Samples were obtained on Day 0, but could be performed up to 7 days prior to the first vaccination, and at the 6-month visit.

### Immunological assay sample allocation

All immunological assays were performed based on sample availability. For each assay, all available samples were used for analysis assuming as sample integrity allowed. For cellular assessments, the ELISpot assay was given priority and remaining cells were then allocated to ICS and/or Lytic Granule Loading assays, followed by the Gene Array assay. TCR sequencing was performed on DNA isolated from Digene brushes so long as the yield from the DNA extraction was sufficient for sequencing purposes.

### ELISA

A standardized binding ELISA was performed to measure the anti–HPV16/18 E6 or E7 antibody response induced by VGX-3100. Endpoint titers of antibodies were determined by coating 96-well enzyme immunoassay plates with 1 mg/ml HPV16 or HPV18 E6 or E7 proteins (recombinant HPV16/18 E7 and HPV16 E6 were procured from ProteinX Lab, San Diego, CA; recombinant HPV18 E6 was cloned and purified from Escherichia coli expression vector at Inovio Pharmaceuticals). Samples were considered positive if the average optical density (OD) of a sample was greater than 0.15 absorbance units and greater than the average OD at baseline (prior to receiving any doses of VGX-3100) plus 2.5 times SD of OD at baseline at the same dilution. For graphing purposes, values of 0 were changed 1 in order to be displayed on a log scale.

### ELISpot

The ELISpot assay was performed by the University of Pennsylvania Human Immunology Core Facility using a qualified protocol as previously described.^1^ The standard ELISpot protocol with 24 hours peptide stimulation was previously cross-validated across different laboratories using HIV-specific peptide pools. Differences between the above-referenced protocol and the protocol used in the current study relate only to the use of HPV peptides as the stimulating antigen (in place of HIV peptides) and not to the length of PBMC stimulation, which remained at 18 to 24 hours per protocol. Specifically, the current protocol used two sets of pooled peptides, each containing 2.5 μg/ml of 15–amino acid residues overlapping by 11 amino acids spanning the full-length consensus sequence of HPV16 E6 and HPV16 E7 or HPV18 E6 and HPV18 E7 antigens. The average number of spot forming units (SFU) counted in R10 wells (media alone) was subtracted from the average in HPV peptide pool stimulated wells and then adjusted to 1 × 10^6^ PBMCs for each HPV peptide pool.

### Intracellular cytokine staining assay

Intracellular cytokine staining was performed as previously described(1) using the following markers: CD3-APCH7, CD107a PECy7, CD14-Pacific Blue, CD16-Pacific Blue, CD4-PECy5.5, IFN-γ-PerCPCy5.5, CD45RO-AF700, CD19-Pacific Blue (BD Biosciences, San Jose, CA), TNFα-AF647, CD8-BV570, CCR7-BV711 (BioLegend, San Diego, CA), granzyme B-PE Texas Red (Invitrogen), CD27-PECy5 (eBioscience, San Diego, CA) perforin-FITC(Abcam, Cambridge, UK). Prepared cells were acquired using an LSR II flow cytometer equipped with BD FACSDiva software (BD Biosciences). Acquired data was analyzed using the FlowJo software version 7.6.3 (Tree Star).

### Lytic granule loading assay

The lytic granule loading assay was performed as previously described1. Briefly, 10^6^ PBMCs were seeded into a 96-well plate. For antigen-specific responses, cells were stimulated 120 hours with HPV16/18 E6 and E7 antigens, while R10 media alone was used as a negative control, OVA was used as an irrelevant peptide control and concanavalin A was used as a positive control (Sigma-Aldrich). At the end of the 5-day incubation period, all samples were washed with phosphate buffered saline and subjected to staining for CD3-APCH7, CD4-PerCPCy5.5, CD8-FITC, CD137-APC (BD Biosciences), Granzyme B-PETR and Perforin-PE using the same protocol as listed above. Staining was performed once per time point per sample listed.

### Gene transcript analysis

PBMCs isolated after the VGX-3100 boost were thawed and rested overnight. After recovery, cells were counted and plated at 200,000 cells per well in a 96-well flat, clear bottom plate. Cells were stimulated with HPV16 E6/E7 and HPV18 E6/E7 pooled peptides (2 µg/ml per well) or received no stimulation (R10 media only). After 21 hours, cells were collected and the total RNA was purified using the PureLink RNA Mini Kit and reverse transcribed to cDNA using the High Capacity cDNA Reverse Transcription Kit (Thermo Fisher). The NanoPhotometer was used to calculate cDNA sample concentrations. TaqMan Array, Human Immune (Thermo Fisher), fast 96-well plates were used to perform RT-qPCR on the QuantStudio 7 Flex Real-Time PCR System using a thermal cycle of 50 °C for 2 minutes, 95 °C for 20 seconds, 40 × 95 °C for 3 seconds, 60 °C for 30 seconds. Data was analyzed using the QuantStudio RT qPCR software. The gene expression heat map was generated using the program software R i386 3.2.0.

### TCR sequencing

PMBC and cervical Digene swab samples used for DNA isolation were obtained from subjects pre- and postboost. PBMC samples from pre- and postboost were stimulated with pooled HPV16 E6/E7 and HPV18 E6/E7 peptides or irrelevant OVA peptides (preboost only) for 5 days prior to isolating DNA for TCR sequence analysis (ImmunoSEQ, Adaptive Biotechnologies). DNA was isolated directly from cervical samples without any stimulation and from negative control cord blood samples after 5 days of stimulation with either HPV or OVA peptides.

## Notes

The following authors are or were employed by Inovio Pharmaceuticals and are compensated as such: M.P.M., K.A.K., A.J.S., X.S., D.A., L.S., J.Y., J.B., C.R., L.H., A.S.K., K.B., K.M-P., M.G., J.L., J.K., N.Y.S., M.L.B. D.B.W. is a member of the Board of Directors for Inovio Pharmaceuticals. C.L.T. has worked with Inovio on other HPV-related clinical trials. The other authors have no conflicts to declare.

## References

[bib1] Stier, EA, Sebring, MC, Mendez, AE, Ba, FS, Trimble, DD and Chiao, EY (2015). Prevalence of anal human papillomavirus infection and anal HPV-related disorders in women: a systematic review. Am J Obstet Gynecol 213: 278–309. 25797230 10.1016/j.ajog.2015.03.034 PMC4556545

[bib2] Boscolo-Rizzo P, Holzinger D (2015). From HPV-positive towards HPV-driven oropharyngeal squamous cell carcinomas. Cancer Treat Rev 42: 24–49. 26547133 10.1016/j.ctrv.2015.10.009

[bib3] Welters MJ, de Vos van Steenwijk PJ, Löwik MJ, Berends-van der Meer DM, Essahsah F, Stynenbosch LF, et al. (2012). Success or failure of vaccination for HPV16-positive vulvar lesions correlates with kinetics and phenotype of induced T-cell responses. Proc Natl Acad Sci USA 107: 11865–11869. 10.1073/pnas.1006500107 PMC2900675 20547850

[bib4] Miller DL and Stack MS. Virology and molecular pathogenesis of HPV (human papillomavirus)-associated oropharyngeal squamous cell carcinoma. Biochem J. 2012;442: 339–353. 10.1042/BJ20112017 PMC3571652 22452816

[bib5] Malik, AI (2005). The role of human papilloma virus (HPV) in the aetiology of cervical cancer. J Pak Med Assoc 55: 553–558. 16438278

[bib6] World Health Organization. Human papillomavirus and HPV vaccines: technical information for policymakers and health professional, 2007. http://wwwwhoint/reproductivehealth/publications/cancers/RHR_08_14/en/indexhtml.

[bib7] Flaherty A, Giuliano A, Magliocco A, Hakky TS, Pagliaro LC, Spiess PE (2014). Implications for human papillomavirus in penile cancer. Urol Oncol 32: 53.e1-8. 10.1016/j.urolonc.2013.08.010 24239463

[bib8] Loizou, C, Laurell, G, Arvidsson, A, Lindquist, D, Nylander, K and Olofsson, K (2015). Recurrent respiratory papillomatosis in northern Sweden: clinical characteristics and practical guidance. Acta Otolaryngol 135: 1058–1064. 26004132 10.3109/00016489.2015.1048378

[bib9] http://www.cdc.gov/std/treatment/2010/hpv.htm.

[bib10] Smith JS, Hoots B, Keys J, Franceschi S, Winer R, Clifford GM (2007). Human papillomavirus type distribution in invasive cervical cancer and high-grade cervical lesions: a meta-analysis update. Int J Cancer 121: 621–632. 17405118 10.1002/ijc.22527

[bib11] Ramqvist, T and Dalianis, T (2010). Oropharyngeal cancer epidemic and human papillomavirus. Emerg Infect Dis 16: 1671–1677. 21029523 10.3201/eid1611.100452 PMC3294514

[bib12] Koskinen, WJ, Chen, RW, Leivo, I, Mäkitie, A, Bäck, L, Kontio, R et al. (2003). Prevalence and physical status of human papillomavirus in squamous cell carcinomas of the head and neck. Int J Cancer 107: 401–406. 14506740 10.1002/ijc.11381

[bib13] 2014. Human papillomavirus in head and neck cancer. Cancers (Basel) 6: 1705–1726. 25256828 10.3390/cancers6031705 PMC4190563

[bib14] Massad LS, Huh WK, Katki HA, Kinney WK, Schiffman M, Solomon D, et al. 2013). 2012 ASCCP Consensus Guidelines Conference. 2012 updated consensus guidelines for the management of abnormal cervical cancer screening tests and cancer precursors. Obstet Gynecol 121: 829–46. 23635684 10.1097/AOG.0b013e3182883a34

[bib15] Kang WD and Kim SM (2015). A HPV-16 or HPV-18 genotype is a reliable predictor of residual disease in a subsequent hysterectomy following a loop electrosurgical excision procedure for cervical intraepithelial neoplasia (CIN) 3. J Gynecol Oncol. 10.3802/jgo.2016.27.e2 PMC4695452 26463431

[bib16] World Health Organization. WHO Guidelines for Screening and Treatment of Precancerous Lesions for Cervical Cancer Prevention. Geneva: World Health Organization. 2013. 24716265

[bib17] Kreimer, AR, Guido, RS, Solomon, D, Schiffman, M, Wacholder, S, Jeronimo, J et al. (2006). Human papillomavirus testing following loop electrosurgical excision procedure identifies women at risk for posttreatment cervical intraepithelial neoplasia grade 2 or 3 disease. Cancer Epidemiol Biomarkers Prev 15: 908–914. 16702369 10.1158/1055-9965.EPI-05-0845

[bib18] Kim, YT, Lee, JM, Hur, SY, Cho, CH, Kim, YT, Kim, SC et al. (2010). Clearance of human papillomavirus infection after successful conization in patients with cervical intraepithelial neoplasia. Int J Cancer 126: 1903–1909. 19642095 10.1002/ijc.24794

[bib19] Xi KN, Wheeler CM, Kreimer A, Ho J, Koutsky LA (2007). Risk of cervical intraepithelial neoplasia grade 2 or 3 after loop electrosurgical excision procedure associated with human papillomavirus type 16 variants. J Infect Dis 195: 1340–1344. 17397005 10.1086/513441

[bib20] Zanussi, S, Simonelli, C, D’Andrea, M, Caffau, C, Clerici, M, Tirelli, U et al. (1996). CD8+ lymphocyte phenotype and cytokine production in long-term non-progressor and in progressor patients with HIV-1 infection. Clin Exp Immunol 105: 220–224. 8706325 10.1046/j.1365-2249.1996.d01-746.x PMC2200507

[bib21] Day CL, Leslie AJ, van der Stok M, Nair K, Ismail N, Honeyborne I, et al. (2007). Proliferative capacity of epitope-specific CD8 T-cell responses is inversely related to viral load in chronic human immunodeficiency virus type 1 infection. J Virol 81: 434–438. 17050606 10.1128/JVI.01754-06 PMC1797250

[bib22] Ward, MJ, Thirdborough, SM, Mellows, T, Riley, C, Harris, S, Suchak, K et al. (2014). Tumour-infiltrating lymphocytes predict for outcome in HPV-positive oropharyngeal cancer. Br J Cancer 110: 489–500. 24169344 10.1038/bjc.2013.639 PMC3899750

[bib23] Hibma, MH. (2012). The immune response to papillomavirus during infection persistence and regression. Open Virol J 6: 241–248. 23341859 10.2174/1874357901206010241 PMC3547310

[bib24] Bagarazzi, ML, Yan, J, Morrow, MP, Shen, X, Parker, RL, Lee, JC et al. (2012). Immunotherapy against HPV16/18 generates potent TH1 and cytotoxic cellular immune responses. Sci Transl Med 4: 155ra138. 10.1126/scitranslmed.3004414 PMC4317299 23052295

[bib25] Trimble CL, Morrow MP, Kraynyak KA, Shen X, Dallas M, Yan J, et al. (2015). Safety, efficacy, and immunogenicity of VGX-3100, a therapeutic synthetic DNA vaccine targeting human papillomavirus 16 and 18 E6 and E7 proteins for cervical intraepithelial neoplasia 2/3: a randomised, double-blind, placebo-controlled phase 2b trial. Lancet. pii: S0140-6736(15)00239-1. doi: 10.1016/S0140-6736(15)00239-1. 2078–2088. 26386540 10.1016/S0140-6736(15)00239-1 PMC4888059

[bib26] Varadarajan N, Yamanaka YJ, Chen H, Ogunniyi AO, McAndrew E, Porter LC, et al. (2011). A high-throughput single-cell analysis of human CD8⁺ T cell functions reveals discordance for cytokine secretion and cytolysis. J Clin Invest 121: 4322–4331. 21965332 10.1172/JCI58653 PMC3204845

[bib27] Migueles SA, Berkley AM, Guo T, Mendoza D, Patamawenu A, Hallahan CW, et al. (2001). Trivalent adenovirus type 5 HIV recombinant vaccine primes for modest cytotoxic capacity that is greatest in humans with protective HLA class I alleles. PLoS Pathog 7. 10.1371/journal.ppat.1002002 PMC3044701 21383976

[bib28] Hagen TL and Eggermont AM (2008). Tumor necrosis factor-mediated interactions between inflammatory response and tumor vascular bed. Immunol Rev 222: 299–315. 18364010 10.1111/j.1600-065X.2008.00619.x

[bib29] Obrador, E, Carretero, J, Pellicer, JA and Estrela, JM (2001). Possible mechanisms for tumour cell sensitivity to TNF-alpha and potential therapeutic applications. Curr Pharm Biotechnol 2: 119–130. 11480417 10.2174/1389201013378743

[bib30] Cullen SP and Martin SJ (2010). Granzymes in cancer and immunity. Cell Death Differ 17: 616–623. 20075940 10.1038/cdd.2009.206

[bib31] Berke, G (1995). The CTL’s kiss of death. Cell 81: 9–12. 7536631 10.1016/0092-8674(95)90365-8

[bib32] Malyguine A, Zaritskaya L, Baseler M, Shafer-Weaver K (2007). New approaches for monitoring CLT activity in clinical trials. Adv Exp Med Biol 601: 273–284. 17713015 10.1007/978-0-387-72005-0_29

[bib33] Betts, MR, Nason, MC, West, SM, De Rosa, SC, Migueles, SA, Abraham, J et al. (2006). HIV nonprogressors preferentially maintain highly functional HIV-specific CD8+ T cells. Blood 107: 4781–4789. 16467198 10.1182/blood-2005-12-4818 PMC1895811

[bib34] Selleck, WA, Canfield, SE, Hassen, WA, Meseck, M, Kuzmin, AI, Eisensmith, RC et al. (2003). IFN-gamma sensitization of prostate cancer cells to Fas-mediated death: a gene therapy approach. Mol Ther 7: 185–192. 12597906 10.1016/s1525-0016(02)00040-0

[bib35] Fluhr, H, Krenzer, S, Stein, GM, Stork, B, Deperschmidt, M, Wallwiener, D et al. (2007). Interferon-gamma and tumor necrosis factor-alpha sensitize primarily resistant human endometrial stromal cells to Fas-mediated apoptosis. J Cell Sci 120(Pt 23): 4126–4133. 18003704 10.1242/jcs.009761

[bib36] Kulkarni, K, Selesniemi, K and Brown, TL (2006). Interferon-gamma sensitizes the human salivary gland cell line, HSG, to tumor necrosis factor-alpha induced activation of dual apoptotic pathways. Apoptosis 11: 2205–2215. 17051336 10.1007/s10495-006-0281-8

[bib37] Zhang, Y, Zhang, Y, Gu, W and Sun, B (2014). TH1/TH2 cell differentiation and molecular signals. Adv Exp Med Biol 841: 15–44. 25261203 10.1007/978-94-017-9487-9_2

[bib38] Parida, S and Mandal, M (2014). Inflammation induced by human papillomavirus in cervical cancer and its implication in prevention. Eur J Cancer Prev 23: 432–448. 24787377 10.1097/CEJ.0000000000000023

[bib39] Weng LK, Catalfamo M, Li Y, Henkart PA. IL-15 is a growth factor and an activator of CD8 memory T cells. Ann N Y Acad Sci. 2002;975:46–56. 12538153 10.1111/j.1749-6632.2002.tb05940.x

[bib40] Hersperger, AR, Martin, JN, Shin, LY, Sheth, PM, Kovacs, CM, Cosma, GL et al. (2011). Increased HIV-specific CD8+ T-cell cytotoxic potential in HIV elite controllers is associated with T-bet expression. Blood 117: 3799–3808. 21289310 10.1182/blood-2010-12-322727 PMC3083297

[bib41] Otterbein, LE, Soares, MP, Yamashita, K and Bach, FH (2003). Heme oxygenase-1: unleashing the protective properties of heme. Trends Immunol 24: 449–455. 12909459 10.1016/s1471-4906(03)00181-9

[bib42] Hofmeyer KA and Zang X (2011). The PD-1/PD-L1 (B7-H1) pathway in chronic infection-induced cytotoxic T lymphocyte exhaustion. J Biomed Biotechnol 2011. 10.1155/2011/451694 PMC3180079 21960736

[bib43] Sakthivel P and Bruder D. Therapeutic intervention in cancer and chronic viral infections: Antibody mediated manipulation of PD-1/PD-L1 interaction. Rev Recent Clin Trials 2011 10–23. 10.2174/157488712799363262 22023178

[bib44] Ingram JT and Zajac AJ (2011). Exhausted CD8 T cells downregulate the IL-18 receptor and become unresponsive to inflammatory cytokines and bacterial co-infections. PLoS Pathog 7. 10.1371/journal.ppat.1002273 PMC3182940 21980291

[bib45] Wolfl, M, Kuball, J, Ho, WY, Nguyen, H, Manley, TJ, Bleakley, M et al. (2007). Activation-induced expression of CD137 permits detection, isolation, and expansion of the full repertoire of CD8+ T cells responding to antigen without requiring knowledge of epitope specificities. Blood 110: 201–210. 17371945 10.1182/blood-2006-11-056168 PMC1896114

[bib46] Wölfl M, Eyrich M, Schlegel PG, Greenberg PD (2008). Use of CD137 to study the full repertoire of CD8+ T cells without the need to know epitope specificities. Cytometry A 73: 1043–1049. 18561198 10.1002/cyto.a.20594 PMC2784669

[bib47] Ye, Q, Song, DG, Poussin, M, Yamamoto, T, Best, A, Li, C et al. (2014). CD137 accurately identifies and enriches for naturally occurring tumor-reactive T cells in tumor. Clin Cancer Res 20: 44–55. 24045181 10.1158/1078-0432.CCR-13-0945 PMC3947326

[bib48] Maldonado, L, Teague, JE, Morrow, MP, Jotova, I, Wu, TC, Wang, C et al. (2014). Intramuscular therapeutic vaccination targeting HPV16 induces T cell responses that localize in mucosal lesions. Sci Transl Med 6: 221ra13. 10.1126/scitranslmed.3007323 PMC4086631 24477000

[bib49] Baden, LR, Liu, J, Li, H, Johnson, JA, Walsh, SR, Kleinjan, JA et al. (2015). Induction of HIV-1-specific mucosal immune responses following intramuscular recombinant adenovirus serotype 26 HIV-1 vaccination of humans. J Infect Dis 211: 518–528. 25165165 10.1093/infdis/jiu485 PMC4318919

[bib50] Robins, HS, Campregher, PV, Srivastava, SK, Wacher, A, Turtle, CJ, Kahsai, O et al. (2009). Comprehensive assessment of T-cell receptor beta-chain diversity in alphabeta T cells. Blood 114: 4099–4107. 19706884 10.1182/blood-2009-04-217604 PMC2774550

[bib51] Söderlund-Strand, A, Kjellberg, L and Dillner, J (2014). Human papillomavirus type-specific persistence and recurrence after treatment for cervical dysplasia. J Med Virol 86: 634–641. 24123176 10.1002/jmv.23806

[bib52] Nagai, Y, Maehama, T, Asato, T and Kanazawa, K (2000). Persistence of human papillomavirus infection after therapeutic conization for CIN 3: is it an alarm for disease recurrence? Gynecol Oncol 79: 294–299. 11063660 10.1006/gyno.2000.5952

[bib53] Wu J, Luo M and Duan Z. Analysis of residual/recurrent disease and its risk factors after loop electrosurgical excision procedure for high-grade cervical intraepithelial neoplasia. Gynecol Obstet Invest 2015 296–301. 26337009 10.1159/000437423

