# Supplementary figures and images for: Augmentation of cellular and humoral immune responses to HPV16 and HPV18 E6 and E7 antigens by VGX-3100
Source: Mol Ther Oncolytics. 2016 Nov 30;3:16025–. doi: 10.1038/mto.2016.25 (PMC5147865; doi:10.1038/mto.2016.25)

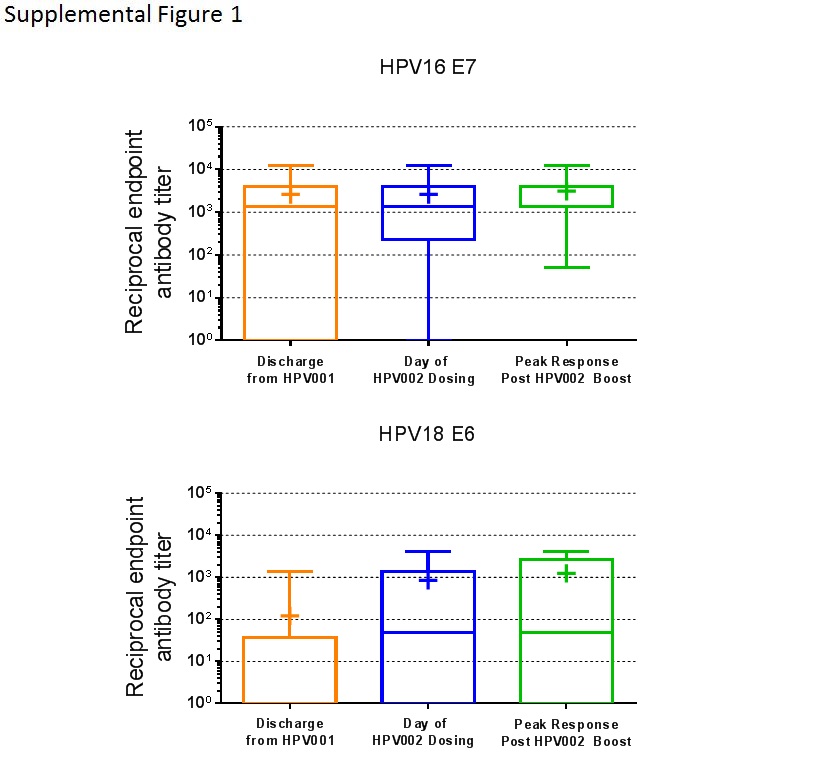

Supplement: Supplementary Figure S1 [file mto201625-s1.jpg]

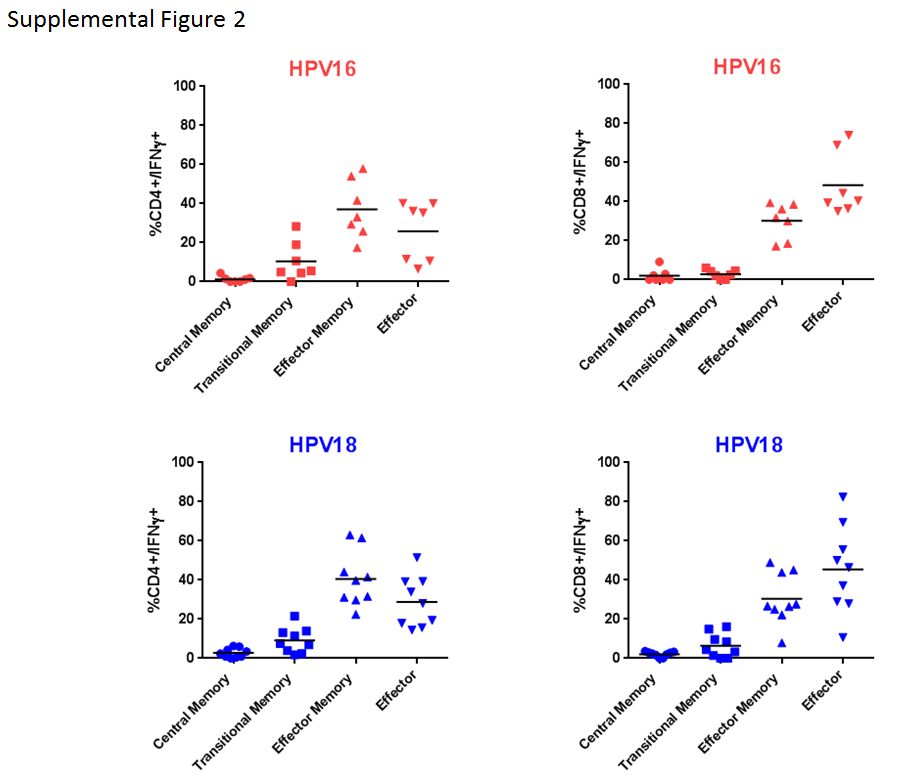

Supplement: Supplementary Figure S2 [file mto201625-s2.jpg]

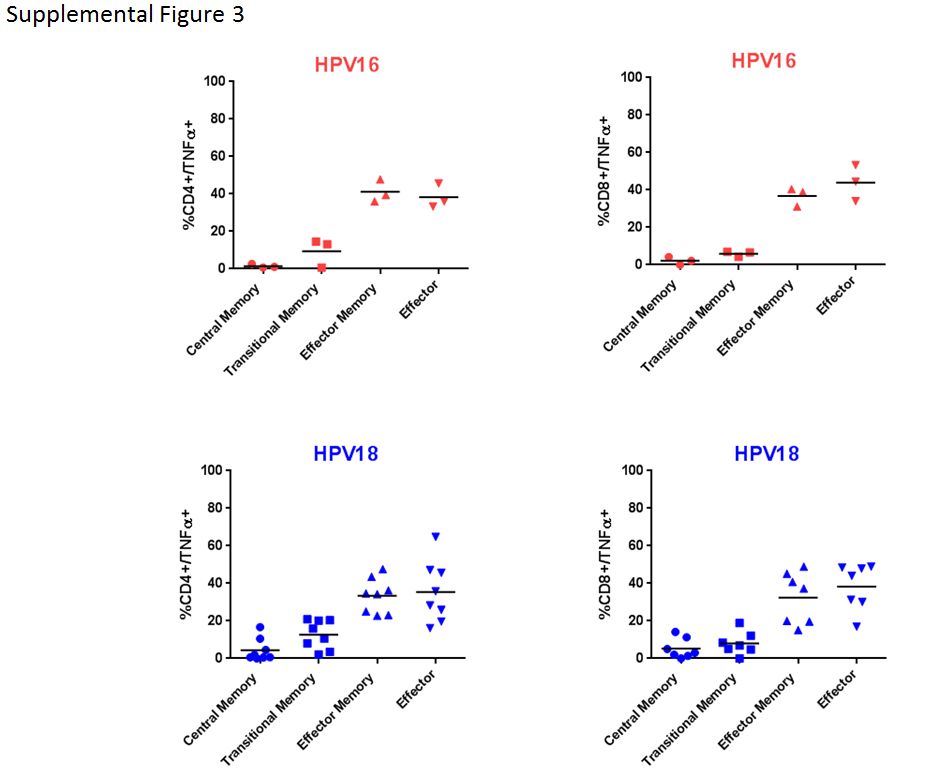

Supplement: Supplementary Figure S3 [file mto201625-s3.jpg]

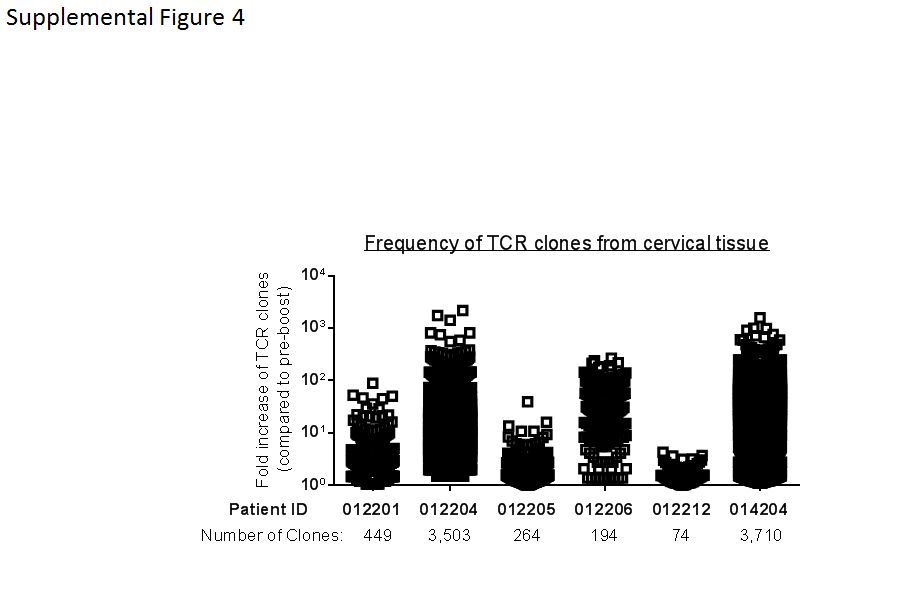

Supplement: Supplementary Figure S4 [file mto201625-s4.jpg]

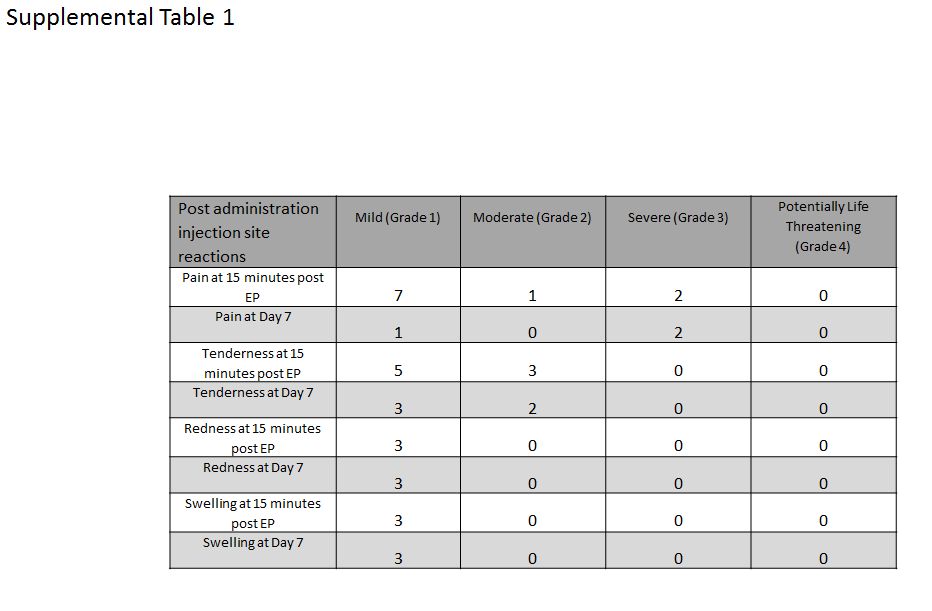

Supplement: Supplementary Table S1 [file mto201625-s5.jpg]

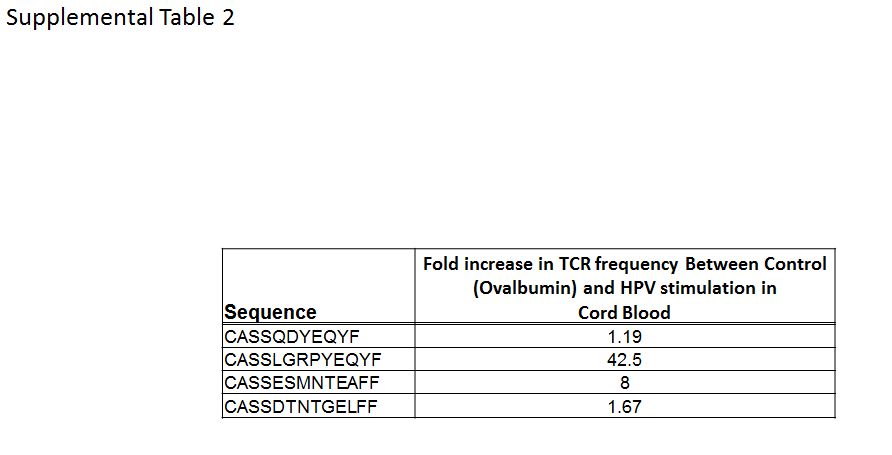

Supplement: Supplementary Table S2 [file mto201625-s6.jpg]

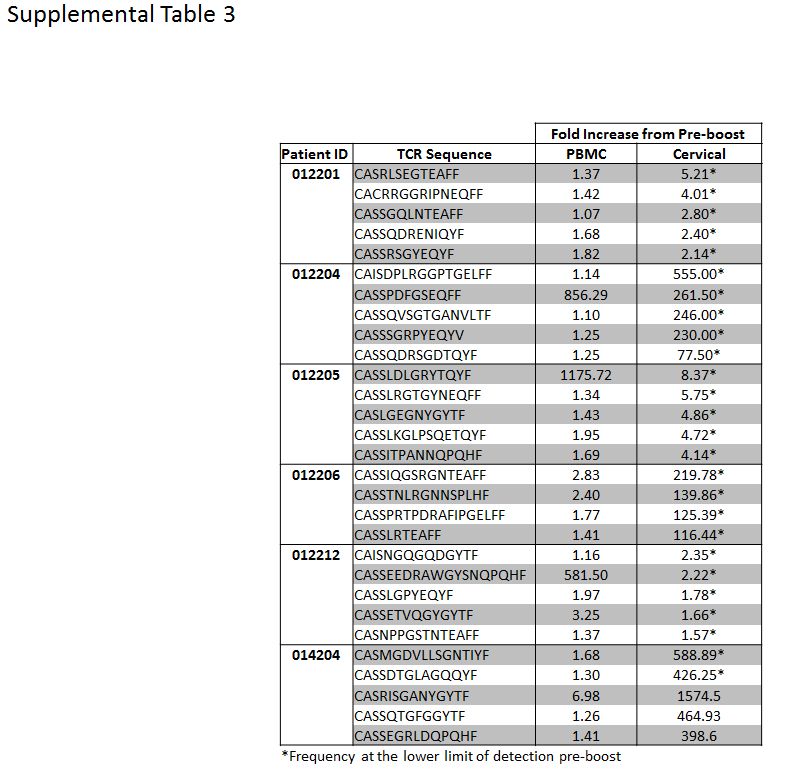

Supplement: Supplementary Table S3 [file mto201625-s7.jpg]

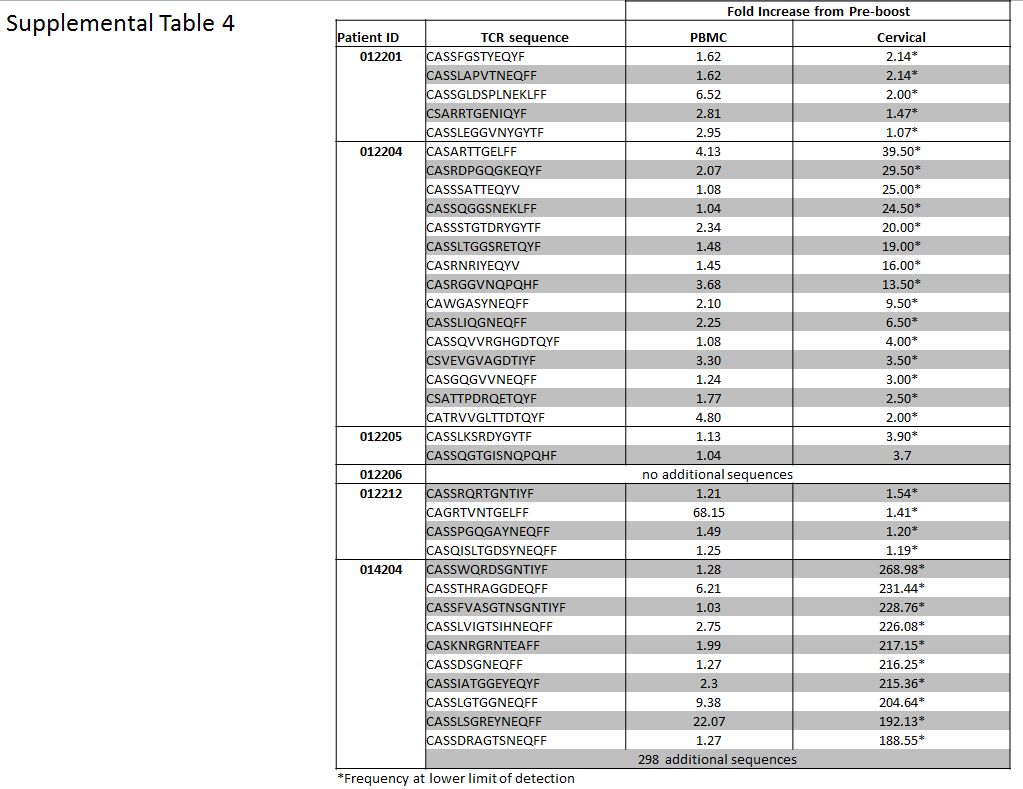

Supplement: Supplementary Table S4 [file mto201625-s8.jpg]
